# Supplementary material for: Hepatitis C Virus Proteins Core and NS5A Are Highly Sensitive to Oxidative Stress-Induced Degradation after eIF2α/ATF4 Pathway Activation
Source: Viruses. 2020 Apr 9;12(4):425. doi: 10.3390/v12040425 (PMC7232227; doi:10.3390/v12040425)
Supplement: Supplementary file 1 [file viruses-12-00425-s001.pdf]

SUPPLEMENTARY FIGURES

A

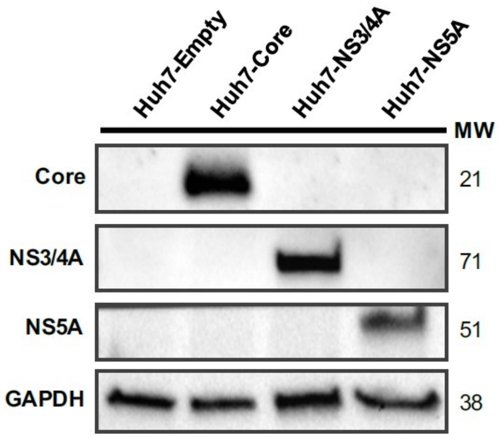

Supplementary Figure S1. HCV Core-, NS3/4A- and NS5A-protein expression. The expression of HCV Core, NS3/4A and NS5A proteins was determined by Western blotting in stably transfected Huh7 cells. The expression of GAPDH was used as a loading control.

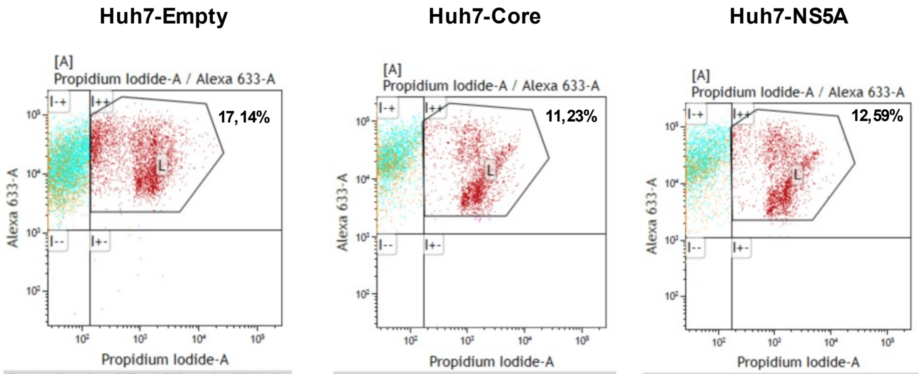

Gate	Number	%Total	%Gated	X-Med
All	12.485	62,43	100,00	53,93
I--	29	0,15	0,23	26,38
I-+	8.957	44,79	71,74	31,63
I+-	8	0,04	0,06	890,82
I++	3.491	17,46	27,96	1.087,49
L	3.427	17,14	27,45	1.117,37

Gate	Number	%Total	%Gated	X-Med
All	12.199	61,00	100,00	26,41
I--	16	0,08	0,13	26,34
I-+	9.928	49,64	81,38	26,37
I+-	0	0,00	0,00	N/A
I++	2.255	11,28	18,49	1.479,52
L	2.245	11,23	18,40	1.477,62

Gate	Number	%Total	%Gated	X-Med
All	11.976	59,88	100,00	26,42
I--	12	0,06	0,10	26,34
I-+	9.433	47,17	78,77	26,37
I+-	0	0,00	0,00	N/A
I++	2.531	12,66	21,13	1.432,88
L	2.518	12,59	21,03	1.432,48

Supplementary Figure S2. Huh7 cells expressing HCV Core and NS5A resist to menadione-induced apoptosis Pre-apoptotic cells were quantified by flow cytometry using DilC(5) (10μM) and PI (100μg/mL) after menadione treatment (50μmol/L)**.**

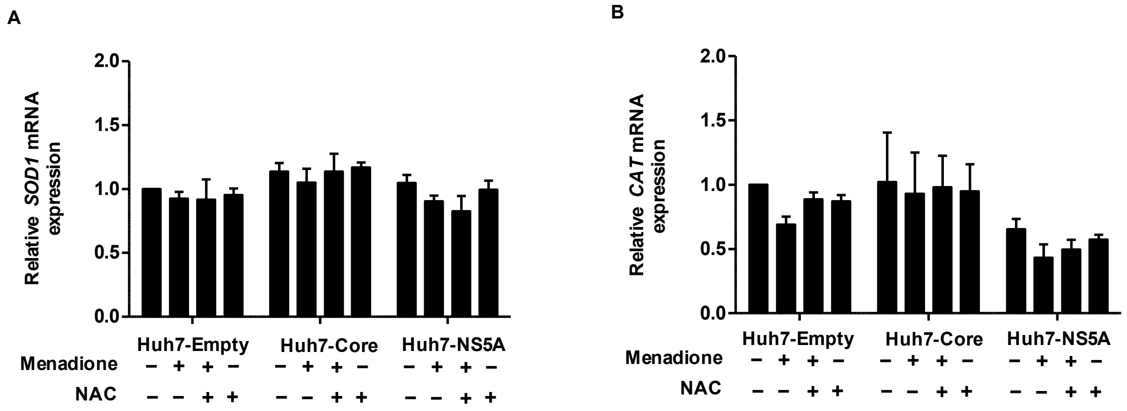

Supplementary Figure S3. Transcriptional regulation of antioxidant enzyme genes is not involved in the protection against menadione-induced apoptosis. The mRNA levels of antioxidant enzymes *SOD1* **(A)** and *CAT* **(B)** were quantified using qPCR in Huh7 cells expressing the empty vector, HCV Core and NS5A. Cells were treated with menadione (50 µmol/L) to induce oxidative stress and in some experiments NAC (5 mmol/L) was used as antioxidant. The relative mRNA expression was normalized relative to 18S. The graphs depict means ± s.d. of three independent experiments. *t* test was performed to compare the means (p values > 0.05 are considered not statistically significant).

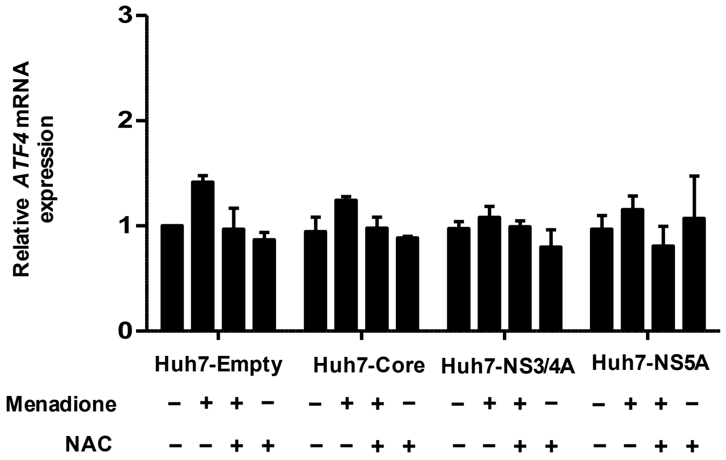

Supplementary Figure S4. ***ATF4*** **mRNA expression.** *ATF4* mRNA expression in Huh7 cells treated with menadione was quantified using qPCR in Huh7 cells expressing the empty vector, HCV Core, NS3/4A and NS5A. Cells were treated with 50 μmol/L menadione and/or 5 mmol/L NAC. The relative expression was normalized relative to 18S. The graphs depict means ± s.d. of three independent experiments. *t* test was performed to compare the means (p values > 0.05 are considered not statistically significant).

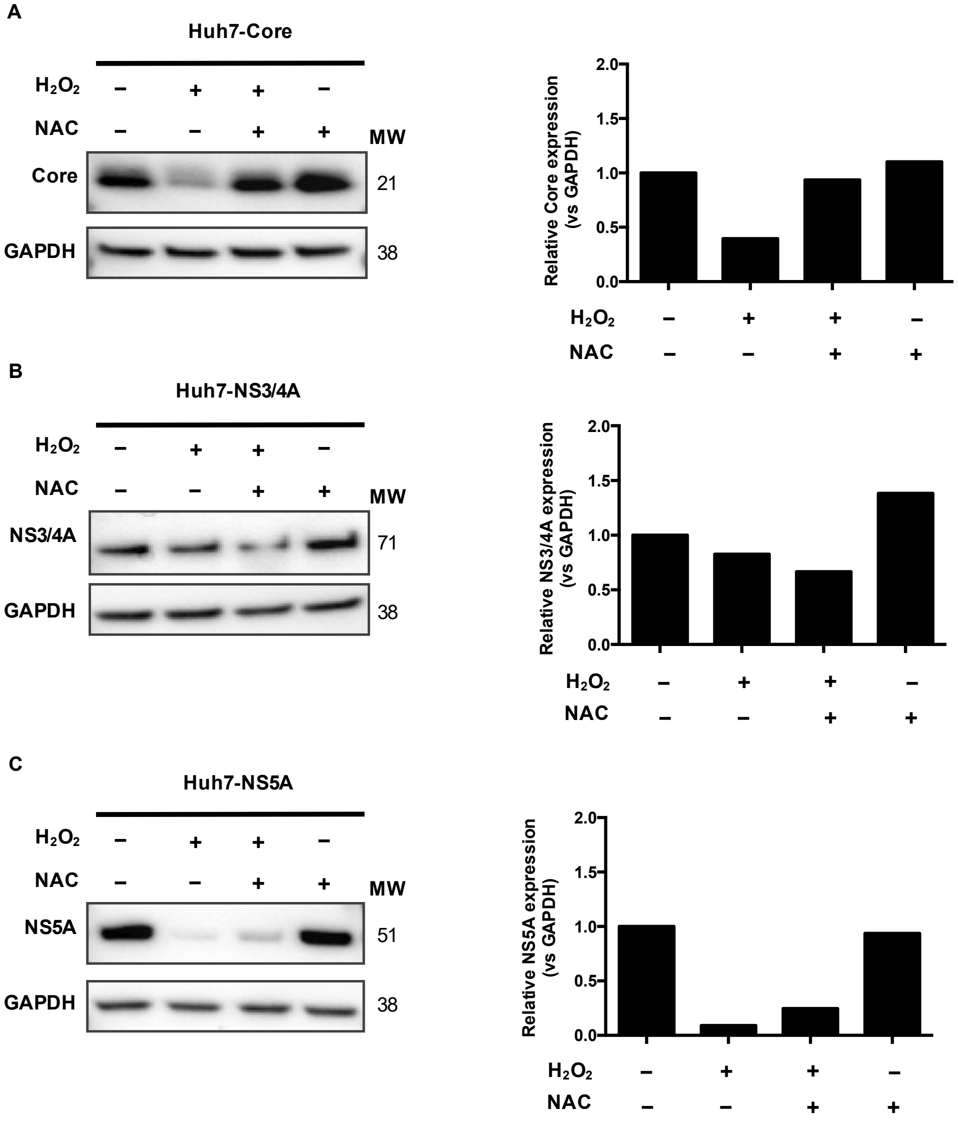

Supplementary Figure S5. Degradation of HCV Core and NS5A is independent of menadione treatment. H_2O_2 (5 mmol/L) was used as an alternative inducer of oxidative stress to confirm the results obtained using menadione. Huh7 cells expressing HCV Core **(A)**, NS3/4A **(B)** and NS5A **(C)** were treated with H_2O_2 for 6 h. In some experiments cells were treated with 5 mmol/L NAC 30 min prior to H_2O_2 treatment. Protein band intensities were quantified using ImageLab software (BioRad). The relative protein expression was calculated based on the expression of GAPDH and compared to the expression of the control cells.

SUPPLEMENTARY TABLES

Supplementary Table 1. Primers and probes for qPCR.

Name	Type	Specie	Sequence (5´- 3´)
**18S_F**	Pr. Forward	Human/Rat	CGG CTA CCA CAT CCA AGG A
**18S_R**	Pr. Reverse	Human/Rat	CCA ATT ACA GGG CCT CGA AA
**18S_P**	Probe	Human/Rat	CGC GCA AAT TAC CCA CTC CCG A
**CuZnSOD_F**	Pr. Forward	Human	CTCACTTTAATCCTCTATCCAGAAAACA
**CuZnSOD_R**	Pr. Reverse	Human	ATCTTTGTCAGCAGTCACATTGC
**CuZnSOD_P**	Probe	Human	CAACATGCCTCTCTTCATCCTTTGGCC
**CAT_F**	Pr. Forward	Human	TTC GAT CTC ACC AAG GTT TGG
**CAT_R**	Pr. Reverse	Human	GTT GCT TGG GTC GAA GGC TAT
**CAT_P**	Probe	Human	CAC AAG GAC TAC CCT CTC ATC CCA GTT GG
**GRP78_F**	Pr. Forward	Human	TGG TGA TCA AGA TAC AGG TGA CCT
**GRP78_R**	Pr. Reverse	Human	GTG TTC CTT GGA ATC AGT TTG GT
**GRP78_P**	Probe	Human	TCC CCT TAC ACT TGG TAT TGA AAC TGT GGG
**ATF4_F**	Pr. Forward	Human	CAG CAA GGA GGA TGC CTT CT
**ATF4_R**	Pr. Reverse	Human	CCA ACA GGG CAT CCA AGT C
**ATF4_P**	Probe	Human	CCA TTT TCT CCA ACA TCC AAT CTG TCC C
**DDIT3_F**	Pr. Forward	Human	GGAAATGAAGAGGAAGAATCAAAAAT
**DDIT3_R**	Pr. Reverse	Human	GTTCTGGCTCCTCCTCAGTCA
**DDIT3_P**	Probe	Human	TTCACCACTCTTGACCCTGCTTCTCTGG

F=Forward/R=Reverse/P=Probe/Pr=Primer
